# Chemotherapy-induced changes in bronchoalveolar lavage fluid CD4 + and CD8 + cells of the opposite lung to the cancer

**DOI:** 10.1038/s41598-020-76752-5

**Published:** 2020-11-16

**Authors:** Georgia Karpathiou, Vasilios Mihailidis, Evangelia Nakou, Stavros Anevlavis, Argyrios Tzouvelekis, George Kouliatsis, Paschalis Ntolios, Demosthenes Bouros, Ioannis Kotsianidis, Marios E. Froudarakis

**Affiliations:** 1 Department of Respiratory Medicine, Medical School of Alexandroupolis, Democritus University of Thrace, Alexandroupolis, Greece; 2 Department of Hematology, Medical School of Alexandroupolis, Democritus University of Thrace, Alexandroupolis, Greece

**Keywords:** Diseases, Medical research, Oncology

## Abstract

Published articles support the effect of chemotherapy in the immune environment of tumors, including lung carcinomas. The role of CD4 + T-cells is crucial for expansion and accumulation of other antigen-specific immune cells, and the participation of CD8 + cells in tumor killing activity has been confirmed by many studies. However, little is known about the effect of chemotherapy on the healthy lung parenchyma from lung cancer patients, and whether there are differences between the different chemotherapy compounds used to treat this patient population. The aim of our study was to explore the effect of chemotherapy on CD4 + and CD8 + cells in the bronchoalveolar lavage fluid (BALF) of the healthy lung in patients treated with standard chemotherapy regimens. Fifteen patients underwent BAL, in the healthy lung before and after six chemotherapy courses. Platinum-based regimens included vinolerbine (VN) in 6 patients, gemcitabine (GEM) in 4 patients and etoposide (EP) in 5 patients. All patients but one were males and smokers (93%). The median age of patients was 56 years (42–75). No significant difference was noted in the patients’ age between the three treated groups. Furthermore, between the three groups, no significant changes in the means of CD4 + and CD8 + cells were noted. However, when we compared the mean CD4 + cells before and after chemotherapy within each group, changes were noted when comparing VN before versus after (*p* = 0.05), GEM before versus after (*p* = 0.03), and EP before versus after (*p* = 0.036). In our pilot study, changes were noted in BALF CD4 + cells for the three most applied regimens at the normal lung parenchyma.

## Introduction

Changes in the local immune microenvirronment play an important role in cancer development. Studies have shown a positive prognostic role of tumor-infiltrating cytotoxic (CD8 +) T lymphocytes in various forms of cancer including lung tumors^1–3^. This ability of the immune system to act against tumor cells is the basis of immunotherapy and the immune system status has a predictive role for this type of therapy. Furthermore, immune system components can be affected by cancer treatments like chemotherapy or radiotherapy, which can act as immunosuppressants reducing lymphocytic counts, or as factors that through necrosis of tumor cells, induce the release of tumor antigens and facilitate the recruitment of immune cells^4^.

The components of the immune system can be evaluated in three different compartments, the peripheral blood, the organs and the tumor itself inside an organ. One well-established method of lung microenvironment investigation is bronchoalveolar lavage (BAL). BAL is a safe non-invasive method that permits one to analyze the local immune response in a large number of lung diseases^5,6^. Studies have shown that lung tumors result in high CD4 + T lymphocyte counts in BAL fluid (BALF) from the affected lung. In addition, after radiation therapy for mammary carcinoma, the increase of lymphocytes found in patients’ BALF mainly consisted of activated cluster of differentiated CD4 + cells migrating from the irradiated lung to the contralateral one^7,8^. Chemotherapy is another cause of lung damage in cancer patients^9^. We have previously shown that early changes of the lung parenchyma may occur without radiology findings in transbronchial biopsies from lung cancer patients treated with chemotherapy^10^. Yet, the effects of chemotherapy in the local immune microenvironment of the opposite lung (to the affected lung) have not been studied in patients with lung cancer, especially with regards to compounds used in a prospective way.

Thus, the aim of this study is to explore possible changes of CD4 + and CD8 + lymphocytes in the BALF of the opposite (healthy) lung after administration of three classic platinum combinations with gemcitabine, etoposide and vinorelbine, used for the treatment of lung cancer patients, in order to assess if these agents can affect the immune microenvironment of the healthy lung.

## Materials and methods

This is a prospective study conducted at the University Hospital, Medical School Democritus University of Thrace after the approval was obtained from the internal Review Board (IRBN 4360/01/11/2007), according to the Greek regulations for good clinical practice. It is part of a research protocol on lung toxicity from chemotherapy and radiation therapy from which two articles have already been published^10,11^. All patients signed informed consent prior to the study. Inclusion criteria were^11^: patients histologically diagnosed with lung cancer, stage IIIB or IV, Eastern Cooperative Oncology Group (ECOG) performance status (PS) 0 or 1, adequate imaging, and no severe comorbidity. Any progression or PS deterioration or serious adverse event including grade III/IV toxicity resulted in the patient’s withdrawal from the study. Finally, 15 patients were included and concluded in the study. They were treated with chemotherapy, prior to radiotherapy, of one of the three platinum-based chemotherapy groups: cisplatin–vinorelbine (VN) (6 patients), cisplatin-gemcitabine (GEM) (4 patients) or cisplatin-etoposide (EP) (5 patients). The first group received 30 mg/m^2^ of VN, administered on days 1 and 8. The second group received 1000 mg/m^2^ of GEM on days 1 and 8 and the third group received 150 mg/m^2^ of EP on days 1–3. All three compounds were given with cisplatin on day 1 at a dose of 100 mg/m^2^. Treatment was repeated in each group every 3 weeks for a total of six planned courses.

**Figure 1:**
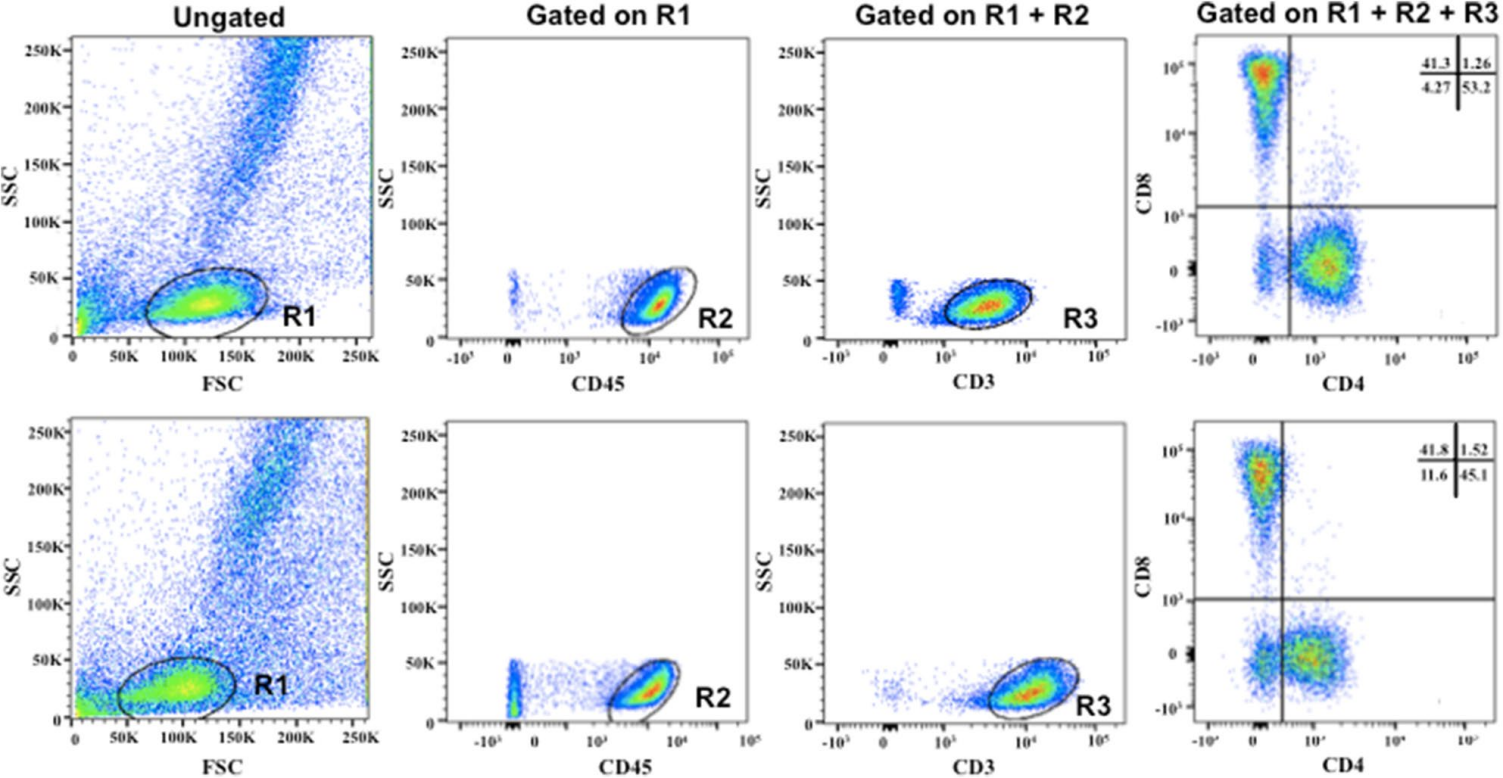
Flow cytometry analysis of BALF in a patient with lung adenocarcinoma before (upper panel) and after (lower panel) “chemotherapy”. Representative flow cytometry plots showing the gating strategy and the percentages of CD4 + and CD8 + T cells.

BAL was conducted in the lung opposite the tumor lung before treatment (T0) during diagnostic bronchoscopy and at the end of six cycles of chemotherapy (T1). During the procedure, 100 ml of 0.9% NaCl solution was instilled at the upper lobe of the contralateral to the tumor lung. The recovery fluid was filtered and centrifuged for 10 min (300 g). For flow cytometry^12^, fresh mononuclear BAL cells were stained with the following antibodies after red cell lysis with ammonium chloride: CD3 (HIT3a), CD4 (RPA-T4), CD8 (RPA-T8), CD45(2D1), and the appropriate isotypic controls, all from BD Pharmingen. Data acquisition and analysis was performed on a FACSCalibur, equipped with CellQuest Pro software (BD biosciences). Treg frequency is reported as percentage of CD4^+^ cells. The cell pellet was used for counting and for flow cytometry with determination of T lymphocyte immunophenotypes after staining with surface anti-CD3 +, anti-CD45, figoanti-CD4 +, and CD8 + antibodies (Fig. 1), as previously described^12^.

Statistical analysis was performed with a statistical software package (StatView version 4.5, Abacus Concepts Inc., Berkeley, CA, USA). CD4 + and CD8 + counts were expressed as mean values ± standard deviation (± SD). Comparison of means between groups was performed with the Student’s *t*-test. The analysis of variances (ANOVA) test was used to determine either whether a compound was a factor of difference (factorial analysis) or the values between the different time points within the same group or different groups (repeated measurements analysis). Statistical significance of all tests was set at a *p* value of less than 0.05.

## Results

Fourteen patients were male (93.3%) and one was female (6.7%). Mean age was 58 years (± 9.4). All patients but one were smokers (93.3%). Ten patients were diagnosed with non-small-cell lung cancer (NSCLC) and five with small-cell lung cancer (SCLC). Patients demographics according to treatment schedule are shown in Table 1^11^.

**Table 1:** Patients characteristics according to the treatment schedule^11^.

	Total	Vinorelbine	Gemcitabine	Etoposide	*p*
Age	58 ± 9.4	61.1 ± 7.8	55 ± 11	56.6 ± 10.9	0.6
Pack-years	76.4 ± 29.4	82 ± 20.2	57.5 ± 46.4	85 ± 20	0.34
**NSCLC**	10 (66.6%)	6	4	0	0.83
ADK	4	1	3		
SqC	4	3	1		
LCC	2	2	0		
SCC	5 (23.3%)			5	
**Stage NSCLC**					0.09
III	7 (46.6%)	3	4		
IV	3 (20%)	3	0		
**Stage SCLC**		0	0		
Limited	4 (26.6%)			5	
Extensive	1 (6.6%)				

**Figure 2:**
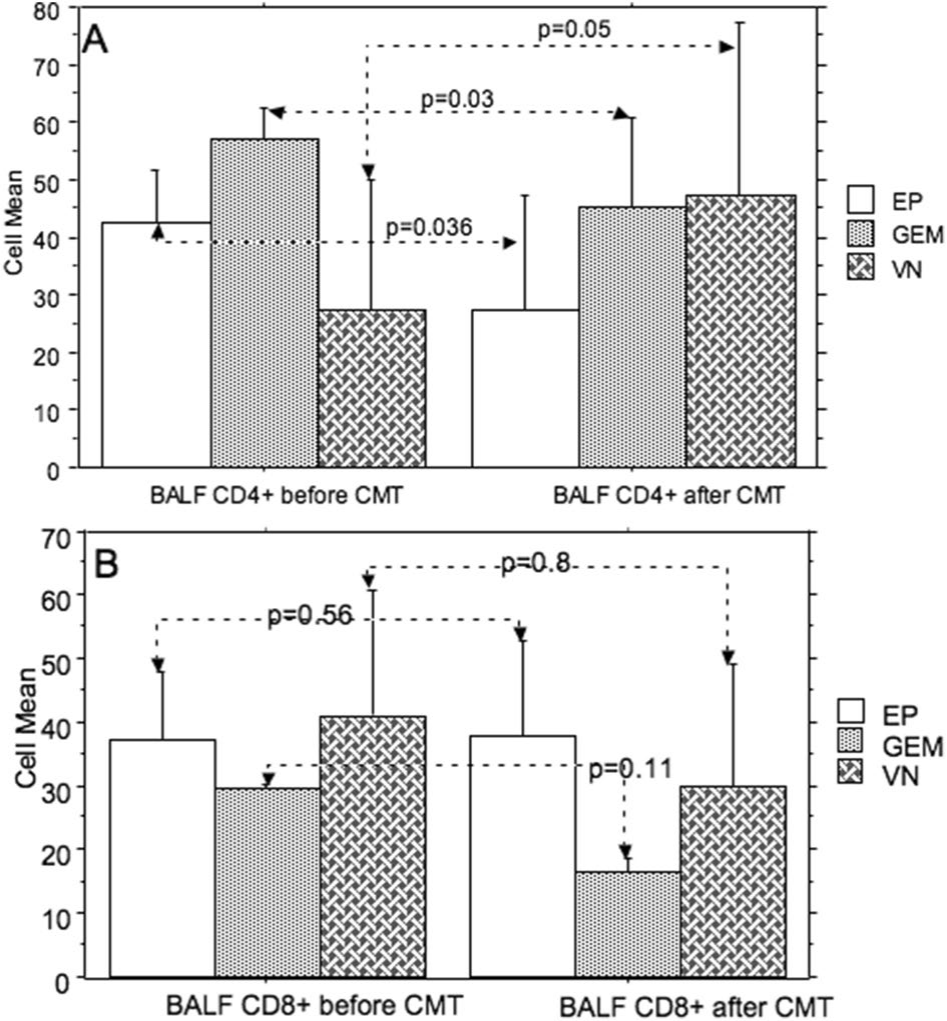
Comparison of (**A**) CD4 + and (**B**) CD8 + mean counts between before (T0) and after (T1) chemotherapy according to the chemotherapy regimen (*EP* etoposide, *VN* vinorelbine, *GEM* gemcitabine—error bars: standard deviations).

CD4 + and CD8 + counts are shown in Table 2: CD4 + and CD8 + counts did not differ significantly between the three chemotherapy groups neither before nor after chemotherapy (Fig. 2). The CD4 + count showed changes after chemotherapy (Table 3) for vinorelbine (*p* = 0.05), gemcitabine (*p* = 0.03), and etoposide (*p* = 0.036), as CD4 + positive lymphocytes increased after VB chemotherapy, while they decreased after GM and EP chemotherapy (Fig. 2). No significant changes were found for CD8 + counts.

**Table 2:** Comparison between the three groups of CD4 + and CD8 + counts (mean ± standard deviation) before (T0) and after (T1) chemotherapy.

	Vinorelbine	Etoposide	Gemcitabine	*p*
CD4 + (T0)	27.5 ± 22.5	42.6 ± 9.2	57 ± 5.6	0.2
CD8 + (T0)	41.1 ± 19.7	37 ± 11	29.5 ± 0.7	0.68
CD4 + (T1)	47.3 ± 30	27.5 ± 19.7	45.2 ± 13	0.41
CD8 + (T1)	30 ± 19	37.6 ± 15	16.3 ± 2	0.24

**Table 3:** Comparison of CD4 + and CD8 + counts (mean ± standard deviation) between before (T0) and after (T1) chemotherapy according to the chemotherapy regimen.

	T0	T1	*p*
**Total**			
CD4 +	39.1 ± 19	39.3 ± 21.3	0.57
CD8 +	37.1 ± 14.1	28 ± 15.3	0.59
**Vinorelbine**			
CD4 +	27.5 ± 22.5	47.3 ± 30	0.05
CD8 +	41.1 ± 19.7	30 ± 1	0.8
**Gemcitabine**			
CD4 +	57 ± 5.6	45.2 ± 13	0.03
CD8 +	29.5 ± 0.7	16.3 ± 2	0.11
**Etoposide**			
CD4 +	42.6 ± 9.2	27.5 ± 19.7	0.036
CD8 +	37 ± 11	37.6 ± 15	0.56

**Figure 3:**
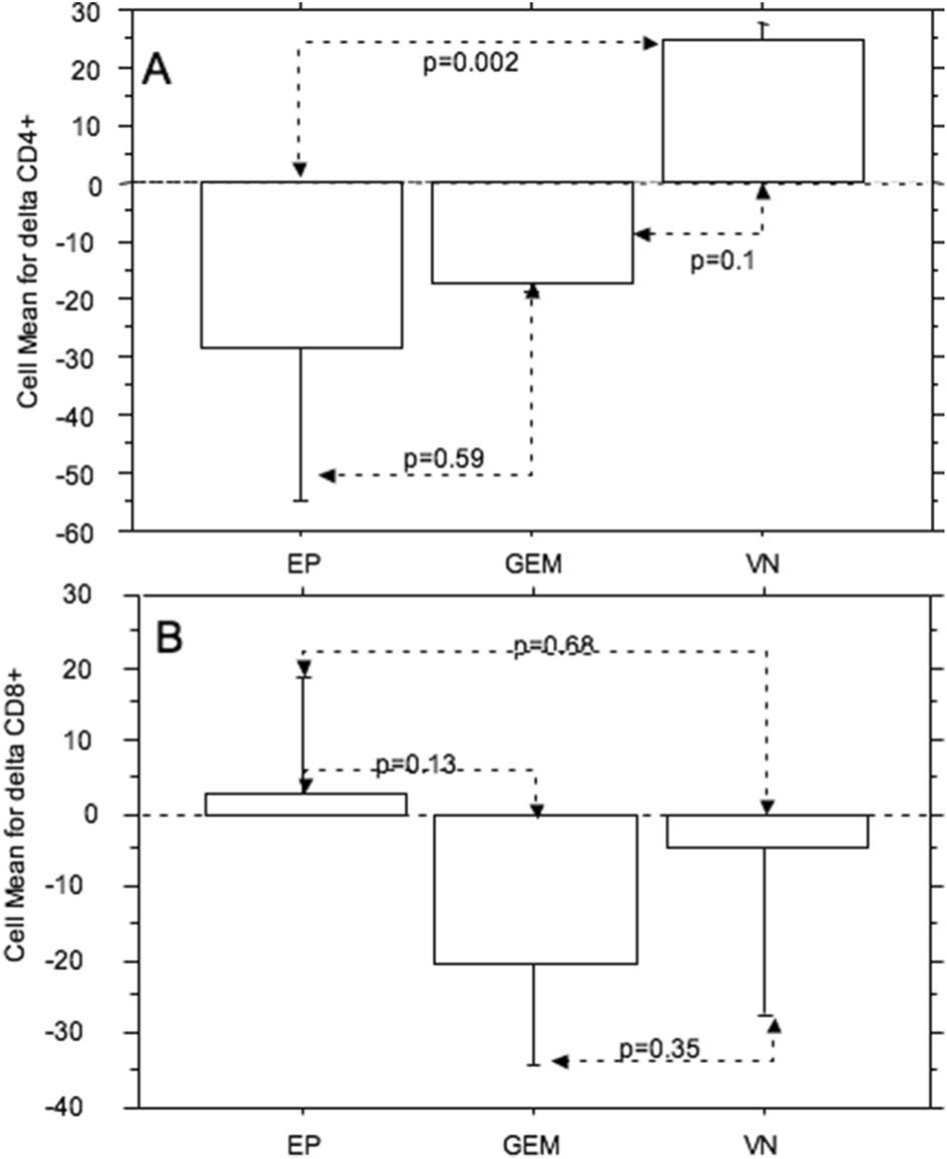
Comparison of the differences (delta) of the values from before to after chemotherapy for CD4 + (**A**) and CD8 + (**B**) mean counts between the chemotherapy regimens (*EP* etoposide, *VN* vinorelbine, *GEM* gemcitabine—error bars: standard deviations).

When the difference (delta) of the value was calculated between the value of the lymphocyte subsets before chemotherapy and the value after chemotherapy and the means were compared between the different groups (EP vs. VN vs. GEM), there was a significant difference between only CD4 + of the VN group and CD4 + of the EP group (*p* = 0.002, Fig. 3).

## Discussion

The literature supports the effect of chemotherapy in the immune environment of the tumors, including lung carcinomas. However, little is known on the effect of chemotherapy on the lung parenchyma opposite the cancer lung, and whether there are specific differences between the different chemotherapy compounds used to treat this patient population. The hypothesis of our study was whether there are differences in the way that various agents act on the lung immune microenvironment that may explain the behavior of the tumor itself regarding these agents. We found that after treatment of three usual platinum combinations in patients with lung cancer, there are differences between gemcitabine (GEM), etoposide (EP) and vinorelbine (VN). It seems that, when added to platinum, VN increases the counts of CD4 + subsets while EP and GEM have the opposite action. In addition, CD8 + subpopulation was decreased in VN and GEM combinations, although it was not statistically significantly, while it remained unchanged in the EP one.

The role of CD4 + helper T-cells is crucial for efficient expansion and accumulation of other antigen-specific immune cells. CD4 + Th1 cells secrete type I cytokines, such as IFN-g and TNF-a, which stimulate CD8 + T cell responses via activation of antigen-presenting cells. CD4 + Th2 cells secrete type II cytokines, such as IL-4, IL-5 and IL-13, which limit the activation of antigen-presenting cells, but may enhance humoral immunity^13^. It is generally considered that Th1^high^/Th2^low^ is a good indicator of an anti-tumor response^13^. In NSCLC, Huang and collaborators^14^ reported that Th1 subsets significantly decrease in tumors, suggesting that the Th1 type immune response is weakened in tumor sites^14^. In our study, although we found that the total number of CD4 + cells did not change after chemotherapy, when we analyzed our subgroups of regimens, the CD4 + cell count in the VN-platinum association increased significantly after chemotherapy, while the CD4 + cell count in GEM and EP combinations decreased. Other investigators reported fewer T-helper cells in tumor infiltrating lymphocytes^15^ or in BALF of patients with lung cancer compared to controls^16^. However, these studies are performed in surgically treated patients with NSCLC, which directly assess the local tumor microenvironment by immunohistochemistry, in terms of prognostic significance^3^, or the BALF of the affected lung^6,17,18^. In addition, the affected pulmonary parenchyma may also suffer from local infection, due to tumoral obstruction, thus, affecting local immunity.

The role of T cytotoxic cells in lung cancer has been well documented. The participation of CD8 + cells in tumor killing activity has been confirmed since the mid-90s by Fujisawa et al.^19^ and Burger et al.^20^. Yoshino and associates^15^ reported an increased proportion of CD8 + cells in tumor infiltrating lymphocytes and Domagala-Kulawik et al.^16^ in BALF, whereas a decrease was observed in the peripheral blood^16^. In our study, the proportion of these cells in the BALF from patients with lung cancer was slightly decreased after chemotherapy. When we looked at our subgroups, this decrease concerned mainly the VN and GEM combinations, while the EP combination remained unchanged. It is also important to consider the influence of cigarette smoking on CD8 + count, which may bias these data. Indeed, Costabel and collaborators in the 1980s^5^ showed an increase in the proportion of these cells in the BALF of smokers.

Lung parenchyma toxicity after chemotherapy also has to be considered when trying to define the tumor immune microenvironment. Bhalla et al.^21^ studied BALF in nine patients with breast cancer receiving four cycles of induction chemotherapy and five healthy controls, showing higher percentages of neutrophils and lymphocytes in the first group. Their study showed that the patients suffered from pulmonary toxicity, according to their lung function tests and the lung inflammatory infiltration, studied by BAL^21^. Similarly, we have previously shown that transbronchial biopsies from the healthy lung parenchyma in patients with lung cancer showed early histological changes after chemotherapy and radiation therapy^10^. This was associated with changes in respiratory function tests^11^. These studies show that the healthy lung parenchyma is influenced by chemoradiotherapy and should be studied separately from the cancer-affected lung.

Parra and associates^22^ studied the tumor tissue microenvironment in patients with NSCLC who underwent neoadjuvant chemotherapy versus no chemotherapy, with further surgical resection. They showed higher densities of CD4 + cells in the pretreated group^22^. Cytotoxic T cells (CD8 +) were assessed by Pasello and investigators^23^ in malignant pleural mesotheliomas (MPM) prior to chemotherapy. Patients who achieved response or stable disease showed lower CD8 + cells compared to those with progressive disease^23^. They reported that higher levels of cytotoxic T lymphocytes remaining after chemotherapy in MPM suggested a more aggressive tumor with lower chances of response to chemotherapy^23^. In rectal cancer, Matsutani et al.^4^ reported that CD8 + lymphocytes in tissue specimens increased after neoadjuvant chemoradiotherapy but not neoadjuvant chemotherapy.

In breast cancer, where neoadjuvant therapy is often the cornerstone of treatment, a decrease of CD4 + T lymphocytes^24^, and an increase of CD8 + T lymphocytes^24,25^ in comparison to the pre-treatment tissue specimen has been reported, suggesting an increase in tumor antigenicity after neoadjuvant chemotherapy^24,25^. Ladoire et al.^26^, on the contrary, did not observe any change in CD8 + infiltrates using a semi-quantitative scoring. Also in breast cancer patients, a 15% decrease in tumor-infiltrating lymphocytes after chemotherapy was reported by Pelekanou et al.^27^.

The reasons for these discrepancies are unclear, but a contribution of chemotherapy regimen cannot be ruled-out, since immune effects differ between different drugs^28^, as was the case in our study. These observations highlight the difficulties in studying the immune microenvironment after treatment because tumor-associated characteristics, treatment differences, or even temporal changes related to treatment schedule, may influence the immune system. In our study, as we are interested in the behavior of the lung opposite the cancer lung, we minimized any tumor-related effect, as by definition this side was not influenced by the tumor. We also minimized this by setting the same time point of investigation at the end of chemotherapy.

Our study has limitations such as the small number of patients enrolled, the lack of subtyping the CD4 + cells, and the absence of follow-up data to compare with overall survival (OS) and progression-free survival (PFS). Indeed, it is difficult to enroll patients with advanced stage lung carcinoma in such a study, even if bronchoscopy is the standard of care for diagnosis and staging the disease, because they will undergo many other investigations and therapeutic interventions during their disease management. In addition, the study was designed to report the differences in T-cell subpopulations and not their prognostic significance with the concern to assess only the microenvironment of the healthy lung parenchyma according to the chemotherapeutic regimen utilized to treat patients with advanced stage lung carcinoma.

To conclude, our pilot study of BAL in the lung parenchyma opposite the cancer lung, showed changes of CD4 + T-lymphocytes, while CD8 + T-lymphocytes showed no significant changes. These results should generate further studies comparing the effect of chemotherapy in healthy lung parenchyma to the effect on tumor microenvironment.

## Data Availability

Data are available upon request.
